# Behavioral motivation support versus personalized dietary management for metabolic and behavioral outcomes in MASLD: a network meta-analysis

**DOI:** 10.1515/med-2026-1542

**Published:** 2026-09-29

**Authors:** Chao Wang, Ziying Huang, Pan Liu, Niuniu Sun, Lanhong Ruan, Mengyuan Qiao

**Affiliations:** School of Nursing, Henan University of Science and Technology, Luoyang, Henan Province, China

**Keywords:** MASLD, network meta-analysis, behavioral intervention, personalized dietary management, self-efficacy

## Abstract

**Objectives:**

MASLD has a prevalence of almost one-third in adults worldwide and is not currently treated pharmacologically with a first-line therapy. Lifestyle change is still the foundation of management; however, the relative effectiveness of behavioral and dietary approaches remains uncertain. We compared behavioral motivation support (BMS), biomarker-guided personalized dietary management (BPDM), general dietary education (GDE), and prescribed dietary models (PDM) with usual care (UC) in adults with MASLD.

**Methods:**

Eight databases and two trial registers were searched from inception to 31 March 2026. The review record was first submitted to PROSPERO on 17 April 2026 and formally registered on 18 April 2026 (CRD420261371973), after the search but before data extraction. Random-effects frequentist NMA was used where networks were sufficiently connected; outcomes measured with different instruments were analyzed using Hedges’ g or narratively.

**Results:**

Fourteen RCTs (n=1,805) were included. Compared with UC, BMS was associated with lower ALT (MD −7.41 U/L, 95 % CI −11.07 to −3.76) and BMI (MD −1.34 kg/m^2^, 95 % CI −2.31 to −0.37), while BPDM was associated with lower ALT (MD −6.72 U/L, 95 % CI −11.76 to −1.68), BMI (MD −1.82 kg/m^2^, 95 % CI −3.15 to −0.50), and body weight (MD −6.66 kg, 95 % CI −8.47 to −4.86). The HbA1c network was sparse and did not include BMS. Nutrition behavior was analyzed as an exploratory two-study pairwise meta-analysis (Hedges’ g 1.64, 95 % CI −0.25 to 3.52), and self-efficacy was synthesized narratively. CINeMA confidence was very low across all 88 assessed network comparisons.

**Conclusions:**

The findings are hypothesis-generating and do not establish superiority, complementary clinical roles, or treatment recommendations. Direct comparative trials are required.

## Introduction

Metabolic dysfunction-associated steatotic liver disease (MASLD), the nomenclature used by the 2023 Delphi multisociety consensus [1], is the most common chronic liver condition globally, with recent epidemiologic syntheses estimating a global adult prevalence of about 30 % [2] and rising in parallel with the obesity and type 2 diabetes mellitus (T2DM) epidemics. The disease progression from simple steatosis to metabolic-associated steatohepatitis (MASH), progressive fibrosis, and ultimately cirrhosis or hepatocellular carcinoma underscores the urgency of effective early intervention. Although resmetirom received regulatory approval in 2024 for selected adults with noncirrhotic MASH and moderate-to-advanced fibrosis, lifestyle modification remains the dominant and most scalable therapeutic option across the broader MASLD population [3], 4]. The evidence base for lifestyle interventions is, however, heterogeneous: calorie-restricted diets, Mediterranean patterns, intermittent fasting regimens, and behavioral counseling programs have each been studied in isolation, yet head-to-head comparisons between strategies remain scarce. Earlier pairwise meta-analyses of dietary interventions demonstrated modest benefits for liver and weight-related outcomes but could not answer which intervention class performs best for which outcome domain [5].

MASLD behavioral and dietary interventions may be generally divided into two dimensions, the level of psychological scaffolding (ranging between minimal general advice to structured behavioral counseling using validated behavior-change theories) and the level of dietary individualization (between standardized guideline-based recommendations to biomarker-driven precision nutrition). Behavioral motivation support (BMS) programs, which include health belief model education, transtheoretical model stage-matching or digital coaching platforms are designed to enhance self-efficacy and maintain long-term adherence through psychological processes. In contrast, personalized diet management based on biomarkers (BPDM) programs make use of individual metabolic profiles, continuous glucose monitoring, or digital-twin modeling to maximize macronutrient prescriptions and calorie targets. General dietary education (GDE) provides a non-individualized but organized nutritional instruction whereas prescribed dietary models (PDM) adopt fixed diets like intermittent fasting or certain macronutrient distributions. The question whether these conceptually different approaches result in meaningfully different effects on the domains of metabolic and behavioral outcomes is not clear.

NMA allows the comparison of several intervention nodes at the same time by combining direct and indirect evidence in a single statistical model, thus overcoming the shortcomings of traditional pairwise meta-analyses that can only compare two interventions simultaneously. Nevertheless, NMA has been rarely applied to behavioral and dietary interventions in MASLD and previous analyses have tended to collapse all the behavioral components into one undifferentiated lifestyle node thereby masking potentially significant between-strategy differences. The current analysis was inspired by the awareness that behavioral motivation programs and precision nutrition approaches are qualitatively different paradigms that have seldom been studied as distinct nodes. We consequently performed a systematic review and NMA of four active classes of interventions (BMS, BPDM, GDE, PDM) vs. usual care (UC) on metabolic outcomes (BMI, body weight, ALT, AST, LSM, HbA1c) and behavioral outcomes (nutrition behavior, self-efficacy), while explicitly examining transitivity, inconsistency, sensitivity to coding decisions, and uncertainty of the evidence.

## Materials and methods

This systematic review and network meta-analysis was conducted and reported in accordance with PRISMA 2020 and the PRISMA extension for network meta-analyses [6], 7]. The review record was first submitted to PROSPERO on 17 April 2026 and formally registered/published on 18 April 2026 (CRD420261371973; version 1.0). The literature search covered database inception to 31 March 2026. Study selection was conducted on 18 April 2026, and data extraction began on 22 April 2026. Registration therefore occurred after completion of the literature search but before data extraction, and the review is not described as prospectively registered.

### Eligibility criteria and search strategy

Eligible studies were RCTs enrolling adults (≥18 years) with confirmed MASLD or NAFLD based on imaging, histology, or validated non-invasive criteria. Any dietary or behavioral modification program within the five prespecified intervention nodes was eligible; usual care, standard lifestyle advice, or placebo-controlled arms served as comparators. Studies were required to report at least one primary or secondary outcome with extractable data. Abstracts-only publications, cross-over designs without washout data, and pediatric studies were excluded. Eight electronic databases (PubMed, Embase, Scopus, Web of Science, WanFang, Cochrane CENTRAL, China National Knowledge Infrastructure [CNKI], and VIP) were searched from inception through 31 March 2026, supplemented by two trial registers (ClinicalTrials.gov and WHO ICTRP). CNKI and VIP were searched using the prespecified strategies but returned no records (both n=0); consequently, no citation-export files were generated for these databases. Searches combined controlled vocabulary and free-text terms covering MASLD/NAFLD, dietary and behavioral interventions, and RCT filters; the full search strategy is provided in Supplementary Table S1. No language restrictions were applied. Records were screened independently in duplicate at both the title/abstract and full-text stages; disagreements were resolved by consensus or consultation with a third reviewer. Data were extracted using a pre-piloted form capturing study design, population characteristics, intervention details, and outcome data.

### Intervention node classification

Five network nodes were defined in advance. UC referred to the trial’s routine-care comparator; comparator intensity was recorded as a transitivity modifier. BMS denoted behavioral motivation support, covering structured interventions that used validated behavior-change techniques, repeated feedback, coaching, or digital self-management support [8], [9], [10], [11], [12], [13], [14], [15], [16]. BPDM represented individualized dietary or lifestyle prescriptions informed by patient-level clinical, metabolic, monitoring, or digital data [17], [18], [19]. GDE referred to structured but non-individualized dietary education [8], 16], 18], and PDM indicated fixed dietary regimens such as 5:2 intermittent fasting or a low-carbohydrate high-fat diet [20], 21]. Mobasheri et al. was recoded from BPDM to BMS because the intervention’s distinguishing component was theory-of-planned-behavior education. The standard-care arm of Joshi et al. was retained as UC; its quarterly physician/nutrition guidance, individualized diet plan, calorie restriction, and exercise prescription were recorded as an enhanced-UC intensity modifier.

### Risk of bias, statistical analysis, and evidence certainty

Risk of bias was assessed at the result level using the Cochrane RoB 2 tool for the effect of assignment to intervention [22]. Signalling-question responses, source/page citations, algorithm guidance, and final domain judgments were retained for 139 NMA results and 10 pairwise or narrative results in Supplementary Table S7. These reconstructed assessments require accountable author verification and are not described as a *de novo* dual-human assessment. Network and pairwise analyses were performed in R 4.5.3 using netmeta 3.4-0 and meta 8.3-0 [23]. Random-effects models used restricted maximum likelihood estimation and a common between-study variance within each outcome network. Continuous outcomes measured on a common scale were expressed as mean differences (MDs), outcomes measured with different instruments as standardized mean differences using Hedges’ g, and binary outcomes as odds ratios (ORs) on the natural OR scale. Outcome direction was harmonized before analysis. We report 95 % confidence intervals, τ^2^, I^2^, Cochran’s Q, prediction intervals when estimable, and p values. Global inconsistency was assessed using the design-by-treatment interaction model and local inconsistency using node-splitting where closed loops existed. P-scores were treated as descriptive secondary outputs and were not generated for networks with four or fewer studies. When SDchange was not reported, it was calculated as √(SDbaseline^2^ + SDfinal^2^ − 2r × SDbaseline × SDfinal), using r=0.5 for the primary calculation and r=0.3 and 0.7 for sensitivity analyses; change means were never combined with final-value SDs [24]. Nutrition behavior was removed from NMA and analyzed as an exploratory two-study pairwise meta-analysis using endpoint Hedges’ g, with change-score analyses at r=0.3, 0.5, and 0.7. Mobasheri’s small dispersion values were converted from SE to SD for the primary reconstruction because that interpretation reproduced the published between-group p value; a literal-published-SD sensitivity analysis was also conducted. Self-efficacy was synthesized narratively without pooling or ranking. Quality of life was analyzed using Hedges’ g with higher scores favorable, while APRI, exercise behavior, and ultrasound normalization were analyzed pairwise. Sensitivity analyses excluded Joshi et al. because its enhanced UC comparator was more intensive than other UC arms. The six CINeMA domains were assessed for each of the 88 estimable intervention-versus-UC network comparisons. Within-study bias and indirectness were based on contribution matrices; reporting bias used the CINeMA labels Suspected/Undetected; imprecision and heterogeneity were judged by comparing confidence and prediction intervals with review-defined equivalence ranges; and incoherence used node-splitting or design-by-treatment results as applicable. Overall confidence started at high and was downgraded without double-counting related concerns. The thresholds were set for this assessment and were not protocol-prespecified universal MCIDs [25]. Research ethics: Not applicable. Informed consent: Not applicable.

## Results

### Study selection

The electronic search retrieved 10,128 records: 10,069 from eight databases (PubMed n=623; Embase n=4,837; Scopus n=784; Web of Science n=965; WanFang n=1,630; Cochrane CENTRAL n=1,230; CNKI n=0; VIP n=0) and 59 from two trial registers (ClinicalTrials.gov n=25; WHO ICTRP n=34). Thus, inclusion of the completed zero-result CNKI and VIP searches changes the database count but not the total number of records. The retained flow records 1,768 duplicates and 1,223 records removed for other reasons, leaving 7,137 records for title/abstract screening; 7,031 were excluded. Of 106 reports and registry records assessed for eligibility, 92 were excluded: ineligible intervention or network (n=52), ineligible population (n=12), same-node comparison (n=12), no usable full-text results/data (n=7), duration <8 weeks (n=7), and protocol-only or duplicate/secondary report (n=2). Supplementary Table S8 lists all 92 eligibility-stage exclusions with reasons. Fourteen studies were included in the quantitative synthesis (Figure 1).

**Figure 1:**
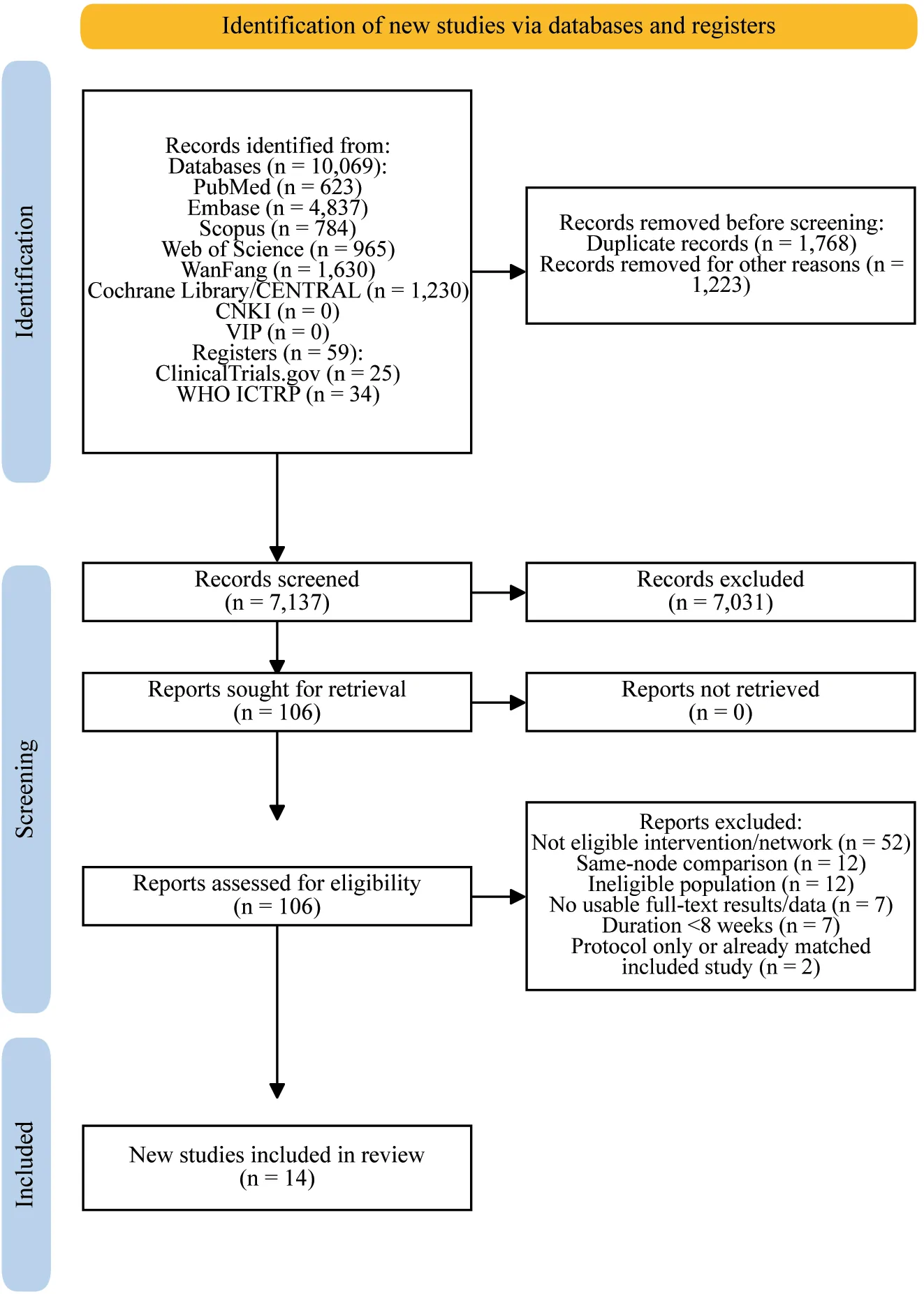
PRISMA 2020 flow diagram of study selection, showing the numbers of records identified, screened, assessed for eligibility, and included in the network meta-analysis.

### Study characteristics

The 14 included randomized controlled trials were published between 2016 and 2026 and involved a total of 1,805 randomized participants from seven countries (Table 1). China contributed six studies, Iran and the United States two each, and Sweden, India, Malaysia, and South Korea one each. Intervention duration ranged from 8 to 104 weeks. The final node mapping included nine original BMS arms, three BPDM arms, three GDE arms, three original PDM arms, and 11 UC arms; the two original Holmer PDM arms were additionally combined into one synthetic analysis arm for node-level synthesis. The principal corrections were Mobasheri (BMS rather than BPDM) and Joshi standard care (UC). Further study- and arm-level characteristics are presented in Supplementary Table S6.

**Table 1:** Characteristics and final intervention-node assignments of the 14 included randomized controlled trials.

Study	Country	Design	Final arm/node mapping (randomized n)	Weeks	Preliminary RoB 2	Classification note
Liu [18]	China	Parallel	ILI (BPDM) vs. GDE; 111/115	12	High	ILI=intensive lifestyle intervention
Holmer [21]	Sweden	Multi-arm	5:2 + LCHF (PDM) vs. UC; 25/25/24	12	High	PDM arms combined for node synthesis
Joshi [19]	India	Parallel	Digital twin (BPDM) vs. enhanced UC; 233/86	52	High	Comparator intensity retained as modifier
Johari [20]	Malaysia	Parallel	Alternate-day calorie restriction (PDM) vs. UC; 33/10	8	High	Pairwise/sparse outcomes
Kwon [16]	South Korea	Parallel	Mobile app coaching (BMS) vs. GDE; 48/54	24	High	Behavioral coaching dominant
Nourian [8]	Iran	Parallel	HBM lifestyle (BMS) vs. GDE; 41/41	8	High	Theory-based behavioral support
Huang [13]	China	Parallel	TLC (BMS) vs. UC; 60/60	12	Some concerns	Repeated behavioral management
Stine [12]	USA	Parallel	Noom mHealth (BMS) vs. UC; 20/20	16	High	Digital coaching
Li [9]	China	Parallel	TTM lifestyle (BMS) vs. UC; 100/100	52	High	Stage-matched support
Sun [17]	China	Parallel	Individualized WeChat program (BPDM) vs. UC; 45/44	24	Some concerns	Patient-level dietary/exercise plan
Axley [11]	USA	Parallel	Text messaging (BMS) vs. UC; 13/17	24	High	Motivational support
Mobasheri [10]	Iran	Parallel	TPB intervention (BMS) vs. UC; 50/50	12	High	Recoded from BPDM to BMS
Dong [14]	China	Parallel	Phone counseling (BMS) vs. UC; 141/139	104	High	Periodic adherence support
Cai [15]	China	Parallel	Multidisciplinary management (BMS) vs. UC; 50/50	12	High	Behavioral coaching and monitoring

BPDM, biomarker-guided personalized dietary management; BMS, behavioral motivation support; GDE, general dietary education; HBM, health belief model; ILI, intensive lifestyle intervention; LCHF, low-carbohydrate high-fat; PDM, prescribed dietary model; TLC, therapeutic lifestyle changes; TPB, theory of planned behavior; TTM, transtheoretical model; UC, usual care.

### Risk of bias in included studies

Formal result-level RoB 2 assessments were available for 139 NMA results: 119 (85.6 %) were judged as having some concerns and 20 (14.4 %) as high risk; none was judged low risk. Among 10 pairwise or narrative results, four had some concerns and six were high risk (Figure 2; Supplementary Table S7).

**Figure 2:**
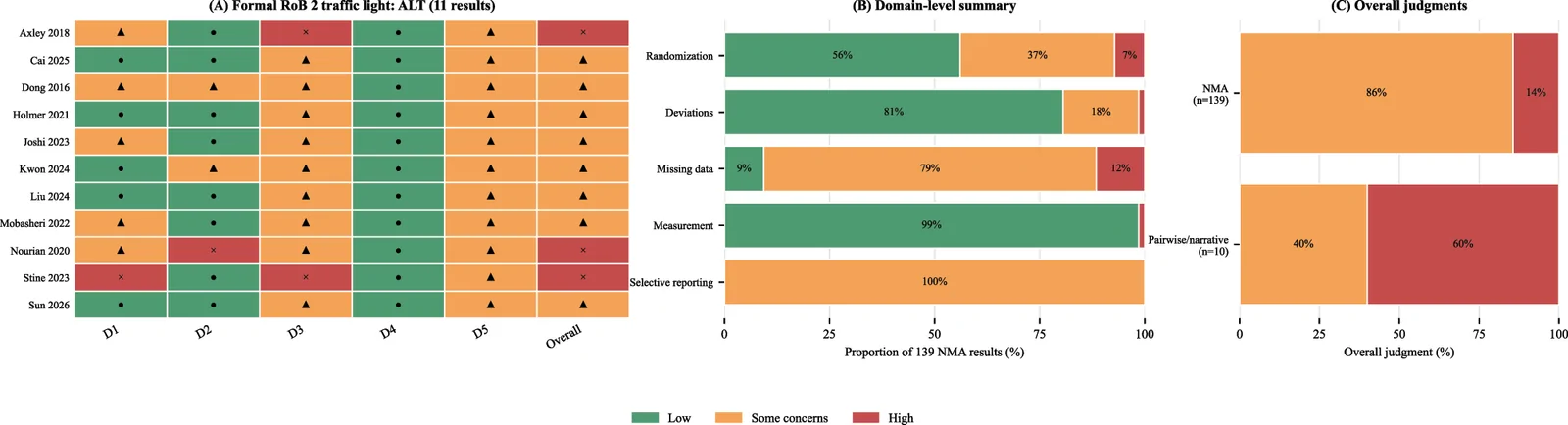
Formal result-level RoB 2 assessment. (A) Traffic-light plot for the 11 ALT study results; (B) domain-level distribution across all 139 NMA results; and (C) overall judgments for 139 NMA results and 10 pairwise or narrative results. Complete result-level assessments are provided in Supplementary Table S7.

For the 139 NMA results, selective reporting was judged as some concerns throughout; missing-outcome-data judgments were some concerns in 79.1 % and high risk in 11.5 %, whereas measurement was low risk in 98.6 %. Figure 2A shows the formal ALT result-level traffic light, Figure 2B summarizes all 139 NMA results by domain, and Figure 2C summarizes the overall judgments for NMA and pairwise or narrative results. The reconstructed assessments retain signalling-question evidence and source/page locations but require accountable human author verification and are not presented as a *de novo* dual-human assessment.

### Network geometry

BMI and ALT each included 11 studies and five nodes, body weight included nine studies and five nodes, and HbA1c included four studies and four nodes. BMS was not represented in the HbA1c network. Figure 3 presents these four core metabolic networks. No trial directly compared BMS with BPDM, and GDE-versus-UC estimates for the core outcomes relied entirely on indirect evidence. Effect-modifier distributions overlapped incompletely, particularly for intervention intensity, contact frequency, individualized dietary planning, duration, and T2DM burden; Joshi’s enhanced UC was therefore examined by exclusion sensitivity analysis (Supplementary Tables S2, S6, and S9).

**Figure 3:**
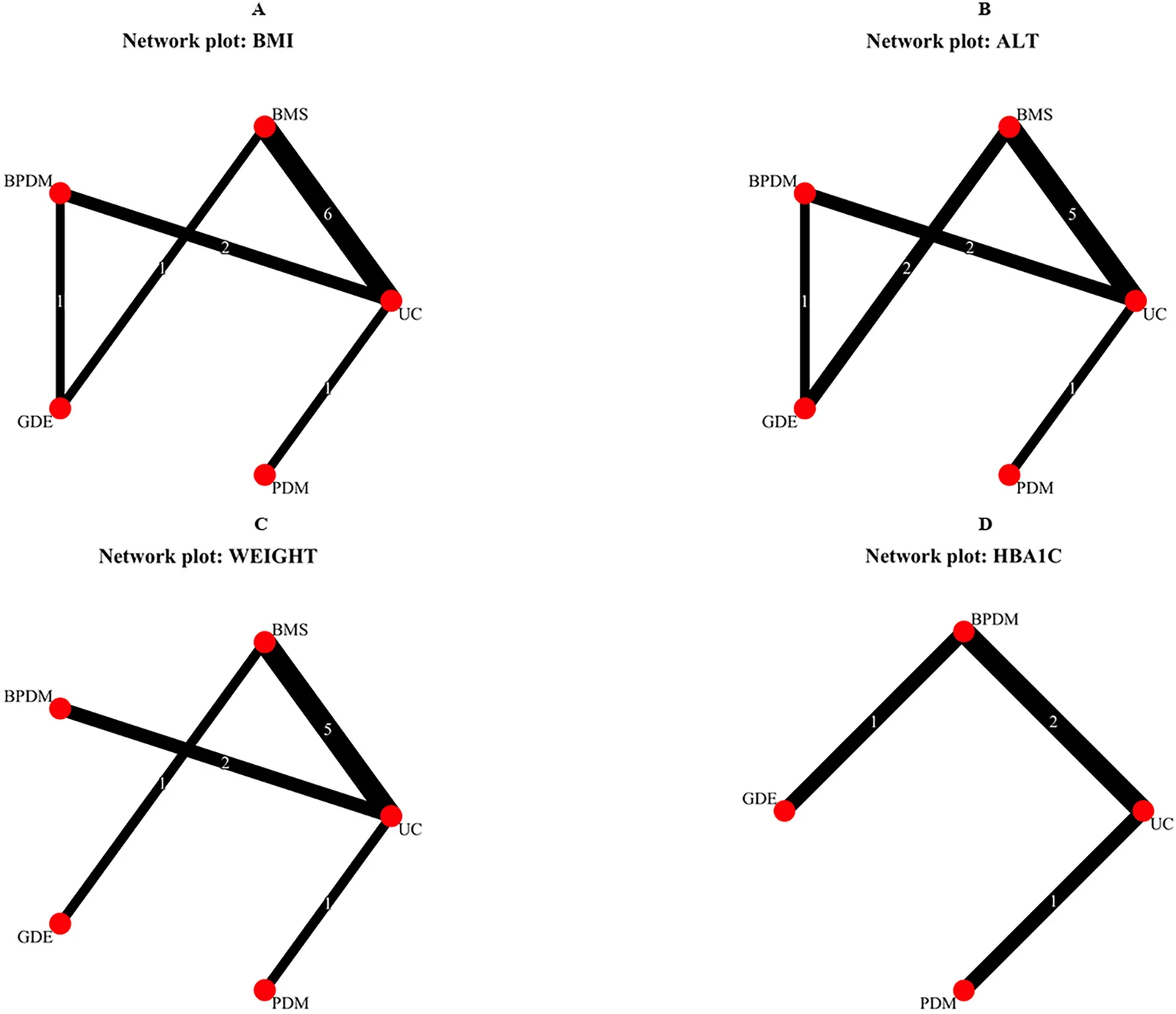
Evidence networks for the four core metabolic outcomes: (A) BMI; (B) ALT; (C) body weight; and (D) HbA1c. Node size is proportional to randomized participants; edge width and labels indicate direct study counts.

### Network meta-analysis results

For ALT (11 studies; 1,299 participants), BMS vs. UC yielded MD −7.41 U/L (95 % CI −11.07 to −3.76; p<0.001) and BPDM vs. UC yielded MD −6.72 U/L (95 % CI −11.76 to −1.68; p=0.009). Network heterogeneity was I^2^=25.5 % (Q p=0.225). Global inconsistency was detected by the design-by-treatment interaction test (p=0.022); the estimable local node-splitting disagreement did not reach statistical significance (p=0.092), although the direct–indirect difference was numerically substantial. AST findings were directionally similar (Supplementary Table S3).

For BMI (11 studies; 1,355 participants), BMS vs. UC yielded MD −1.34 kg/m^2^ (95 % CI −2.31 to −0.37; p=0.007) and BPDM vs. UC MD −1.82 kg/m^2^ (95 % CI −3.15 to −0.50; p=0.007). Heterogeneity was substantial (I^2^=73.8 %; Q p<0.001), but neither global (p=0.860) nor local inconsistency (p=0.869) was detected. For body weight (nine studies; 1,072 participants), BPDM vs. UC yielded MD −6.66 kg (95 % CI −8.47 to −4.86; p<0.001), PDM vs. UC MD −4.85 kg (95 % CI −7.16 to −2.55; p<0.001), and BMS vs. UC MD −1.86 kg (95 % CI −3.96 to 0.24; p=0.082); I^2^ was 0 % (Q p=0.503). The HbA1c network (four studies; 641 participants) was sparse and extremely heterogeneous (I^2^=99.5 %; Q p<0.001); BMS was absent and no ranking was produced. BPDM vs. UC yielded MD −1.31 percentage points (95 % CI −3.83 to 1.21; p=0.308), GDE vs. UC MD −1.16 (95 % CI −5.52 to 3.20; p=0.602), and PDM vs. UC MD −0.27 (95 % CI −3.83 to 3.29; p=0.882). For LSM, BMS vs. UC yielded MD −0.31 (95 % CI −0.76 to 0.14; p=0.177), BPDM −0.32 (95 % CI −1.08 to 0.44; p=0.409), GDE −0.11 (95 % CI −0.96 to 0.75; p=0.803), and PDM 0.42 (95 % CI −0.80 to 1.64; p=0.502); meaningful I^2^ and ranking were not estimable in this sparse network (Supplementary Table S4).

Nutrition behavior was not analyzed using raw MD or NMA. The exploratory two-study endpoint analysis using Hedges’ g yielded g=1.64 (95 % CI −0.25 to 3.52; p=0.058; I^2^=10.7 %). Change-score sensitivity analyses remained imprecise at r=0.3 (g=1.35, 95 % CI −1.63 to 4.33), r=0.5 (g=1.58, 95 % CI −1.91 to 5.07), and r=0.7 (g=2.01, 95 % CI −2.38 to 6.39). Treating Mobasheri’s printed values literally as SDs produced an unstable pooled g=5.55 (95 % CI −43.32 to 54.43; I^2^=99.0 %). Self-efficacy was synthesized narratively because the instruments assessed non-exchangeable constructs. Individual endpoint mean differences were 0.20 points (95 % CI −0.37 to 0.77; p=0.488) in Kwon and 4.95 points (95 % CI 3.10 to 6.80; p<0.001) in Nourian; no pooled effect was calculated. Quality of life was harmonized using Hedges’ g with higher scores favorable; BMS vs. UC was g=1.38 (95 % CI 0.97 to 1.79; p<0.001) and GDE vs. UC g=1.11 (95 % CI 0.55 to 1.68; p<0.001), with no ranking. Exploratory pairwise analyses are also reported for APRI, exercise behavior, and ultrasound normalization (Supplementary Tables S4–S5 and Supplementary Figure S27).

The prespecified subgroup analyses by intervention duration and T2DM burden were not feasible because the strata contained too few studies. Small-study-effect tests were not interpreted for outcomes lacking a sufficient number of independent studies (Figure 4 and Table 2).

**Figure 4:**
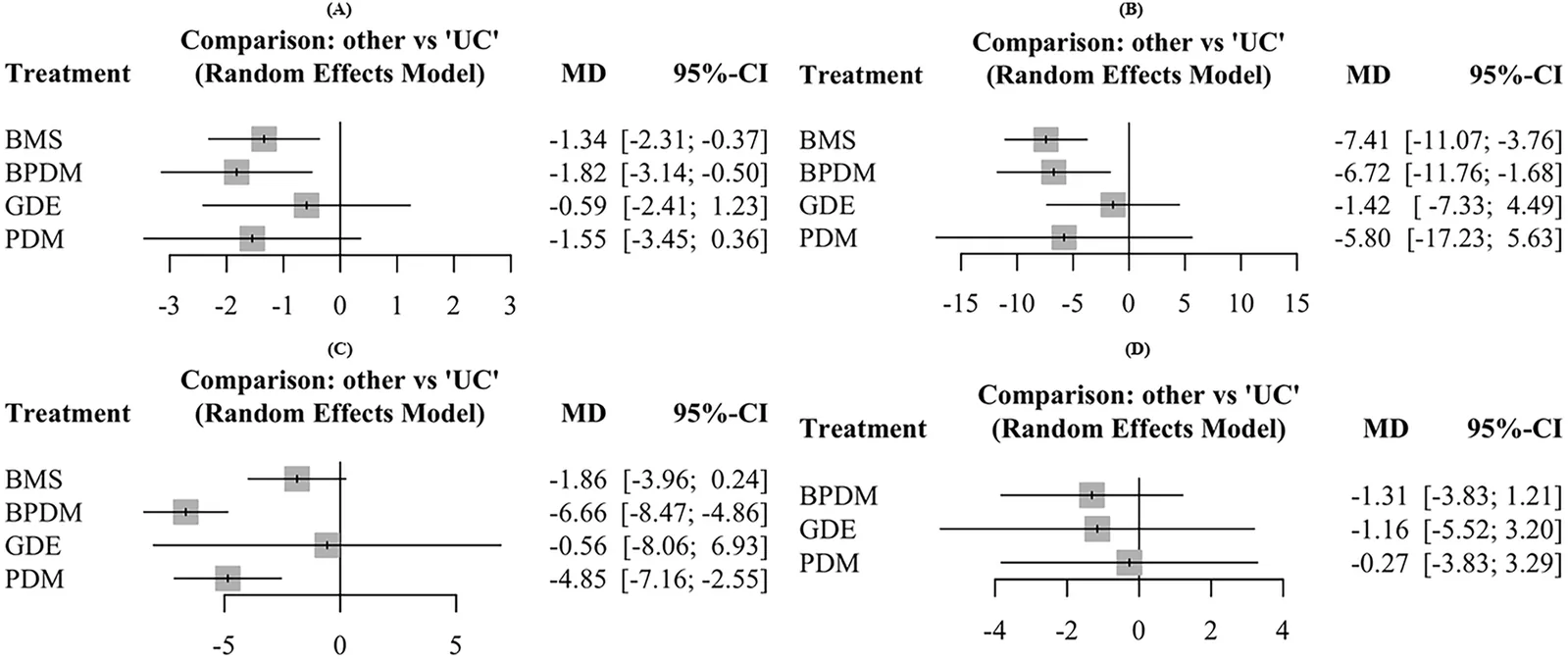
REML random-effects network estimates vs. usual care for (A) BMI; (B) ALT; (C) body weight; and (D) HbA1c. Lower values favor the intervention. HbA1c is a sparse exploratory network and is not ranked.

**Table 2:** Core network meta-analysis estimates versus usual care.

Outcome	Intervention	Effect (95 % CI)	p	Studies (participants)	Ranking status	Heterogeneity and inconsistency
ALT	BMS	MD −7.41 (95 % CI −11.07 to −3.76)	<0.001	11 (1,299)	P-score 0.79 (descriptive)	I^2^ 25.5 %; Q p 0.225; Global p 0.022; local p 0.092
ALT	BPDM	MD −6.72 (95 % CI −11.76 to −1.68)	0.009	11 (1,299)	P-score 0.73 (descriptive)	I^2^ 25.5 %; Q p 0.225; Global p 0.022; local p 0.092
ALT	GDE	MD −1.42 (95 % CI −7.33 to 4.49)	0.637	11 (1,299)	P-score 0.24 (descriptive)	I^2^ 25.5 %; Q p 0.225; Global p 0.022; local p NA
ALT	PDM	MD −5.80 (95 % CI −17.23 to 5.63)	0.320	11 (1,299)	P-score 0.61 (descriptive)	I^2^ 25.5 %; Q p 0.225; Global p 0.022; local p NA
BMI	BMS	MD −1.34 (95 % CI −2.31 to −0.37)	0.007	11 (1,355)	P-score 0.62 (descriptive)	I^2^ 73.8 %; Q p<0.001; Global p 0.860; local p 0.869
BMI	BPDM	MD −1.82 (95 % CI −3.14 to −0.50)	0.007	11 (1,355)	P-score 0.81 (descriptive)	I^2^ 73.8 %; Q p<0.001; Global p 0.860; local p 0.869
BMI	GDE	MD −0.59 (95 % CI −2.41 to 1.23)	0.524	11 (1,355)	P-score 0.32 (descriptive)	I^2^ 73.8 %; Q p<0.001; Global p 0.860; local p NA
BMI	PDM	MD −1.55 (95 % CI −3.45 to 0.36)	0.111	11 (1,355)	P-score 0.67 (descriptive)	I^2^ 73.8 %; Q p<0.001; Global p 0.860; local p NA
Body weight	BMS	MD −1.86 (95 % CI −3.96 to 0.24)	0.082	9 (1,072)	P-score 0.41 (descriptive)	I^2^ 0.0 %; Q p 0.503; Global p NA; local p NA
Body weight	BPDM	MD −6.66 (95 % CI −8.47 to −4.86)	<0.001	9 (1,072)	P-score 0.96 (descriptive)	I^2^ 0.0 %; Q p 0.503; Global p NA; local p NA
Body weight	GDE	MD −0.56 (95 % CI −8.06 to 6.93)	0.883	9 (1,072)	P-score 0.28 (descriptive)	I^2^ 0.0 %; Q p 0.503; Global p NA; local p NA
Body weight	PDM	MD −4.85 (95 % CI −7.16 to −2.55)	<0.001	9 (1,072)	P-score 0.74 (descriptive)	I^2^ 0.0 %; Q p 0.503; Global p NA; local p NA
HbA1c	BPDM	MD −1.31 (95 % CI −3.83 to 1.21)	0.308	4 (641)	Not ranked	I^2^ 99.5 %; Q p<0.001; Global p NA; local p NA
HbA1c	GDE	MD −1.16 (95 % CI −5.52 to 3.20)	0.602	4 (641)	Not ranked	I^2^ 99.5 %; Q p<0.001; Global p NA; local p NA
HbA1c	PDM	MD −0.27 (95 % CI −3.83 to 3.29)	0.882	4 (641)	Not ranked	I^2^ 99.5 %; Q p<0.001; Global p NA; local p NA

P-scores are descriptive secondary outputs and must not be interpreted as head-to-head superiority. Global p denotes the design-by-treatment interaction test; local p denotes node-splitting where estimable. Blank local tests indicate that no relevant closed-loop comparison was estimable.

### Inconsistency, ranking, and certainty of evidence

Excluding Joshi’s enhanced-UC trial changed statistical significance for seven network comparisons, and a separate GDE-versus-UC HbA1c estimate changed direction; these eight material changes indicate sensitivity to comparator intensity rather than robustness (Supplementary Table S9). Figure 5 therefore presents P-scores only descriptively for the better-connected ALT, BMI, and body-weight networks. The six-domain CINeMA assessment covered all 88 estimable intervention-versus-UC network comparisons, including all four body-weight comparisons, and rated confidence very low throughout; Figure 5D and Table 3 display 15 selected core comparisons. Downgrading reflected combinations of within-study bias, suspected reporting bias, and outcome-specific concerns about indirectness, imprecision, heterogeneity, or incoherence. Review-defined clinical decision thresholds were used for this assessment and were not treated as protocol-prespecified universal MCIDs (Table 3; Supplementary Table S10).

**Figure 5:**
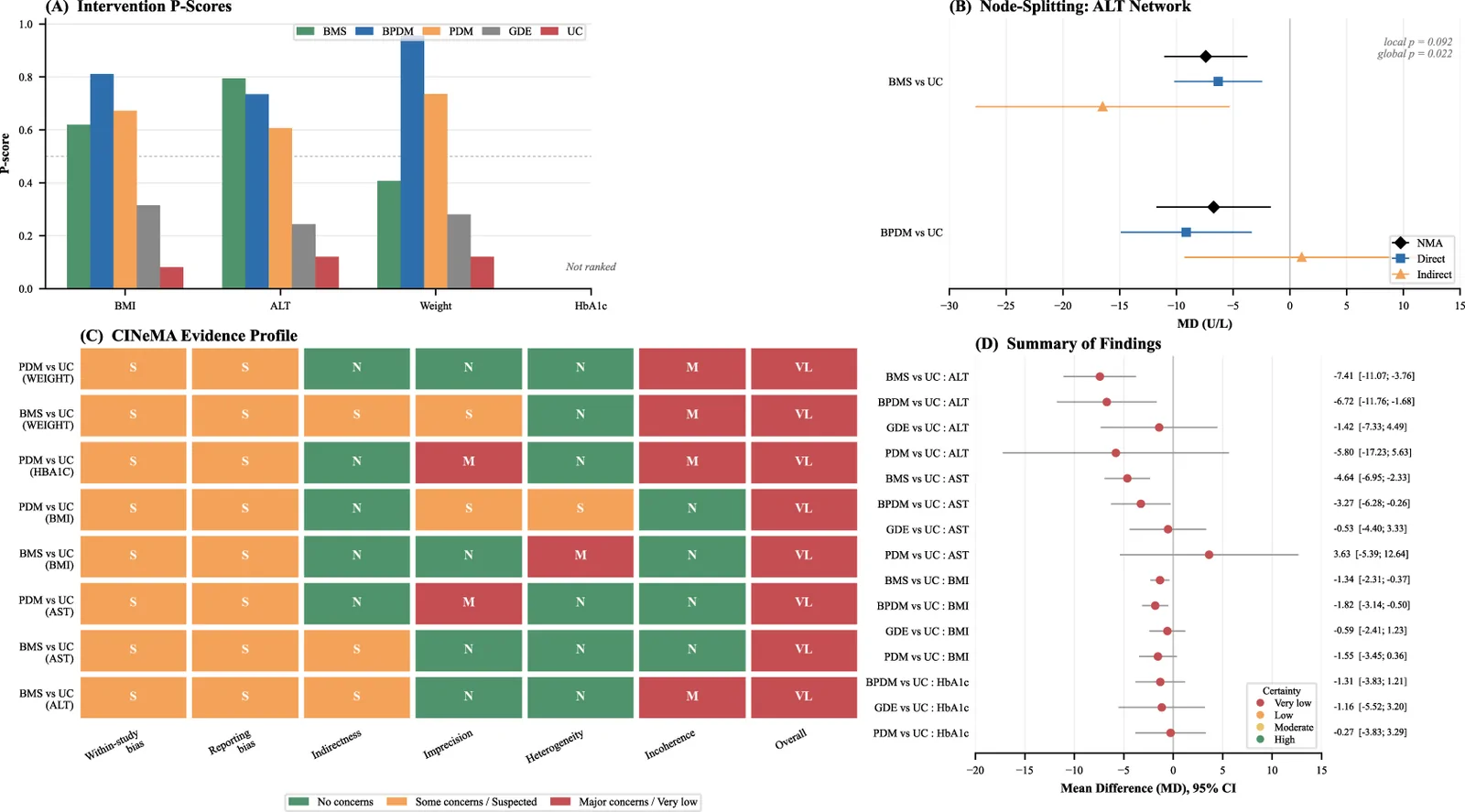
Ranking and certainty of evidence across selected outcomes. (A) Descriptive intervention P-scores for BMI, ALT, and weight; HbA1c was not ranked. (B) Node-splitting results for the ALT network. (C) CINeMA evidence profiles for selected comparisons. (D) Summary of findings for ALT, AST, BMI, and HbA1c. P-scores do not establish clinical superiority; all displayed CINeMA confidence ratings were very low.

**Table 3:** CINeMA six-domain assessment for the 15 comparisons displayed in Figure 5D.

Comparison	REML effect (95 % CI)	Within-study bias/reporting bias/indirectness	Imprecision/heterogeneity/incoherence	Overall status
ALT – BMS vs. UC	MD −7.41 (95 % CI −11.07 to −3.76)	Some concerns/Suspected/Some concerns	No concerns/No concerns/Major concerns	Very low
ALT – BPDM vs. UC	MD −6.72 (95 % CI −11.76 to −1.68)	Some concerns/Suspected/Some concerns	No concerns/Some concerns/Some concerns	Very low
ALT – GDE vs. UC	MD −1.42 (95 % CI −7.33 to 4.49)	Some concerns/Suspected/No concerns	Some concerns/Some concerns/Major concerns	Very low
ALT – PDM vs. UC	MD −5.80 (95 % CI −17.23 to 5.63)	Some concerns/Suspected/No concerns	Major concerns/No concerns/Major concerns	Very low
AST – BMS vs. UC	MD −4.64 (95 % CI −6.95 to −2.33)	Some concerns/Suspected/Some concerns	No concerns/No concerns/No concerns	Very low
AST – BPDM vs. UC	MD −3.27 (95 % CI −6.28 to −0.26)	Some concerns/Suspected/No concerns	No concerns/Some concerns/No concerns	Very low
AST – GDE vs. UC	MD −0.53 (95 % CI −4.40 to 3.33)	Some concerns/Suspected/No concerns	Some concerns/Some concerns/No concerns	Very low
AST – PDM vs. UC	MD 3.63 (95 % CI −5.39 to 12.64)	Some concerns/Suspected/No concerns	Major concerns/No concerns/No concerns	Very low
BMI – BMS vs. UC	MD −1.34 (95 % CI −2.31 to −0.37)	Some concerns/Suspected/No concerns	No concerns/Major concerns/No concerns	Very low
BMI – BPDM vs. UC	MD −1.82 (95 % CI −3.14 to −0.50)	Some concerns/Suspected/Major concerns	No concerns/Some concerns/No concerns	Very low
BMI – GDE vs. UC	MD −0.59 (95 % CI −2.41 to 1.23)	Some concerns/Suspected/Some concerns	Major concerns/No concerns/No concerns	Very low
BMI – PDM vs. UC	MD −1.55 (95 % CI −3.45 to 0.36)	Some concerns/Suspected/No concerns	Some concerns/Some concerns/No concerns	Very low
HbA1c – BPDM vs. UC	MD −1.31 (95 % CI −3.83 to 1.21)	Some concerns/Suspected/No concerns	Major concerns/No concerns/Major concerns	Very low
HbA1c – GDE vs. UC	MD −1.16 (95 % CI −5.52 to 3.20)	Some concerns/Suspected/No concerns	Major concerns/No concerns/Major concerns	Very low
HbA1c – PDM vs. UC	MD −0.27 (95 % CI −3.83 to 3.29)	Some concerns/Suspected/No concerns	Major concerns/No concerns/Major concerns	Very low

All 88 assessed network comparisons were rated very low; Table 3 displays 15 selected core comparisons. Complete six-domain judgments for all 88 comparisons, including body weight, together with prediction intervals, direct-evidence proportions, reporting-bias considerations, and review-defined clinical decision thresholds are detailed in Supplementary Table S10. The thresholds were set for this assessment and should not be interpreted as protocol-prespecified universal MCIDs.

## Discussion

The revised analyses show signals favoring BMS or BPDM over UC for selected outcomes, but the magnitude and, for some comparisons, the direction of effect remain uncertain. BMS and BPDM were both associated with lower ALT and BMI, whereas BPDM had the largest body-weight estimate. BMS was absent from the HbA1c network, and the remaining HbA1c estimates were extremely heterogeneous and imprecise. Nutrition behavior no longer supports a network ranking: its two-study Hedges’ g estimate crossed the null, and self-efficacy could only be described narratively [26], 27].

These analyses do not establish that BMS and BPDM have complementary clinical roles. No head-to-head comparison was available, so cross-node contrasts depend on indirect paths and transitivity assumptions. Intervention and comparator intensity varied, particularly in Joshi, and exclusion of that trial changed statistical significance for seven comparisons and separately reversed one HbA1c estimate. BMI showed substantial heterogeneity but no global or local inconsistency, whereas ALT showed significant global inconsistency with a non-significant local test. Descriptive P-scores therefore cannot justify selecting one intervention over another.

Strengths of this review include the search of eight databases and two trial registers without language restrictions, explicit node definitions, outcome-appropriate effect measures, sensitivity analyses for change-score correlations and uncertain SD/SE labeling, separation of sparse pairwise outcomes from formal NMA, and transparent reporting of the six CINeMA domains.

Several limitations should be considered. PROSPERO registration occurred after the literature search, although before data extraction, so the review cannot be regarded as fully prospectively registered. The result-level RoB 2 and CINeMA assessments were reconstructed from the available reports and project files; clinical decision thresholds were defined for this assessment rather than prespecified in the protocol. Several networks were sparse or heterogeneous; effect-modifier balance was incomplete; Mobasheri’s dispersion labeling was uncertain; and no trial directly compared BMS with BPDM. Follow-up was often short, and small-study-effect assessments were underpowered. Future trials should directly compare clearly defined behavioral-support and personalized-dietary strategies, standardize behavioral and quality-of-life instruments, report change-score variances, document comparator intensity, and assess hepatic, metabolic, behavioral, and patient-reported outcomes over at least 12 months.

## Conclusions

This review provides hypothesis-generating estimates only. The observed outcome-specific signals cannot establish superiority, complementary clinical roles, or treatment recommendations because BMS and BPDM were not compared head to head; several estimates were sparse, heterogeneous, inconsistent, or sensitive to comparator coding; and CINeMA confidence was very low across the assessed network comparisons. Direct, adequately powered trials with standardized outcomes and complete prospective reporting are required before clinical translation.

## Supplementary Material

Supplementary Material

Supplementary Material

Supplementary Material

Supplementary Material

Supplementary Material

Supplementary Material

Supplementary Material

Supplementary Material

Supplementary Material

Supplementary Material

Supplementary Material

Supplementary Material

Supplementary Material
