# Supplementary material for: Behavioral motivation support versus personalized dietary management for metabolic and behavioral outcomes in MASLD: a network meta-analysis
Source: Open Med (Wars). 2026 Sep 29;21(1):20261542. doi: 10.1515/med-2026-1542 (PMC13621874; doi:10.1515/med-2026-1542)
Supplement: Supplementary file 1 [file j_med-2026-1542_suppl_002.pdf]

## A. Endpoint Hedges' g

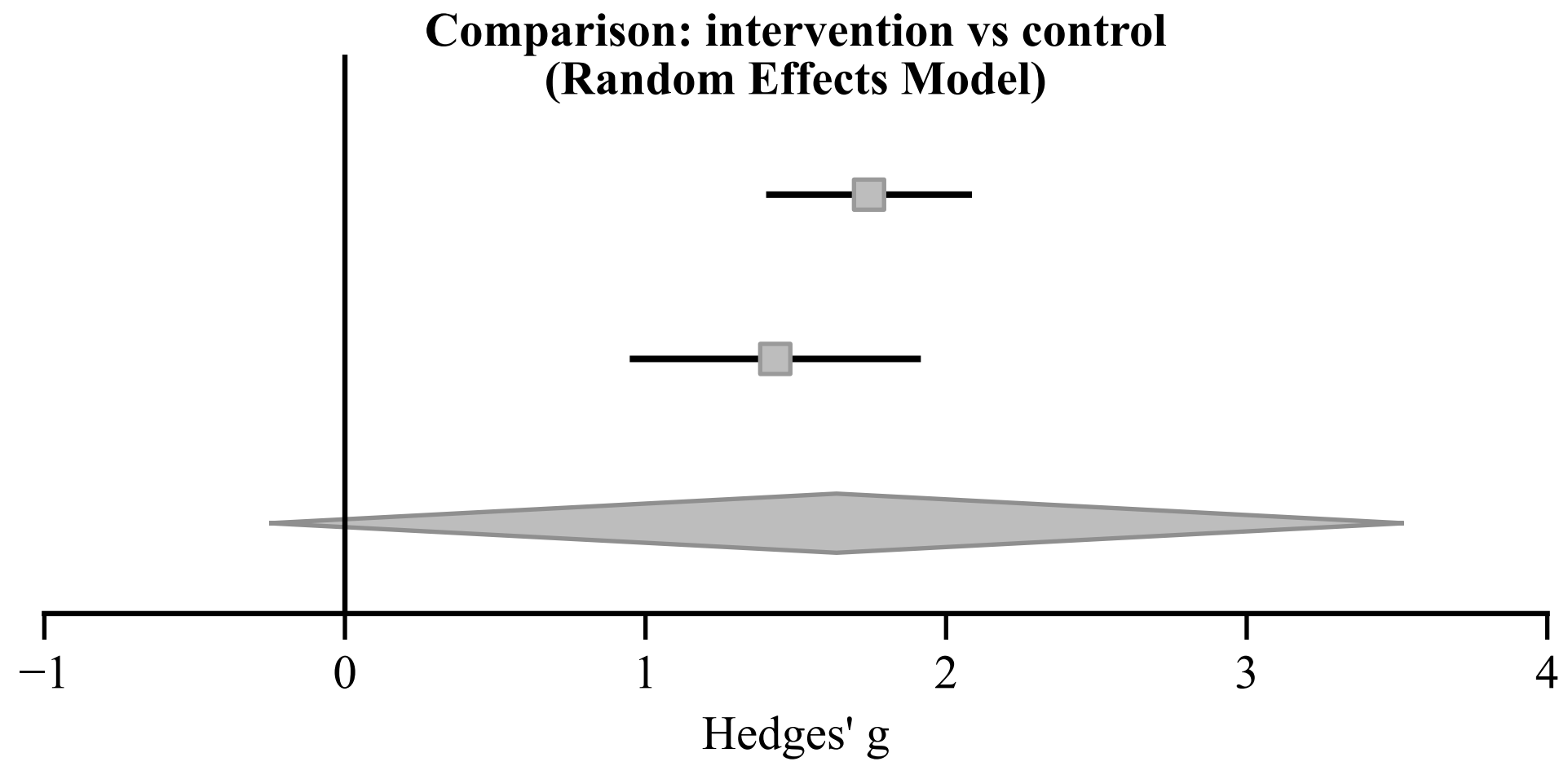


| Study | SMD | 95%-CI |
|---|---|---|
| Li 2022 | 1.74 | [1.41; 2.08] |
| Mobasheri 2022 | 1.43 | [0.96; 1.91] |
| **Pooled** | **1.64** | **[-0.25; 3.52]** |

## B. Change-score Hedges' g (r = 0.5)

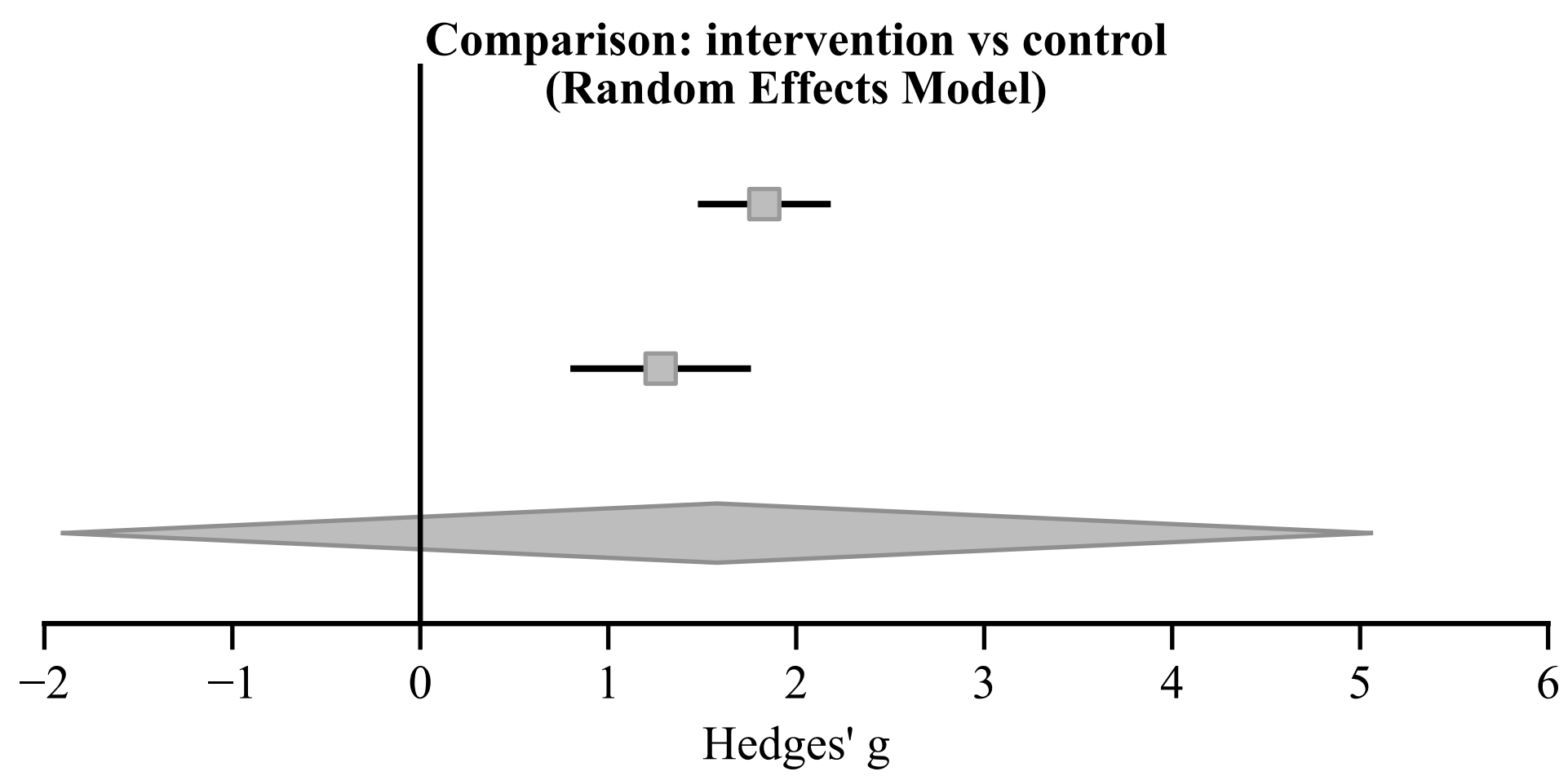


| Study | SMD | 95%-CI |
|---|---|---|
| Li 2022 | 1.83 | [1.49; 2.17] |
| Mobasheri 2022 | 1.28 | [0.82; 1.74] |
| **Pooled** | **1.58** | **[-1.91; 5.07]** |

## C. Literal published-SD sensitivity

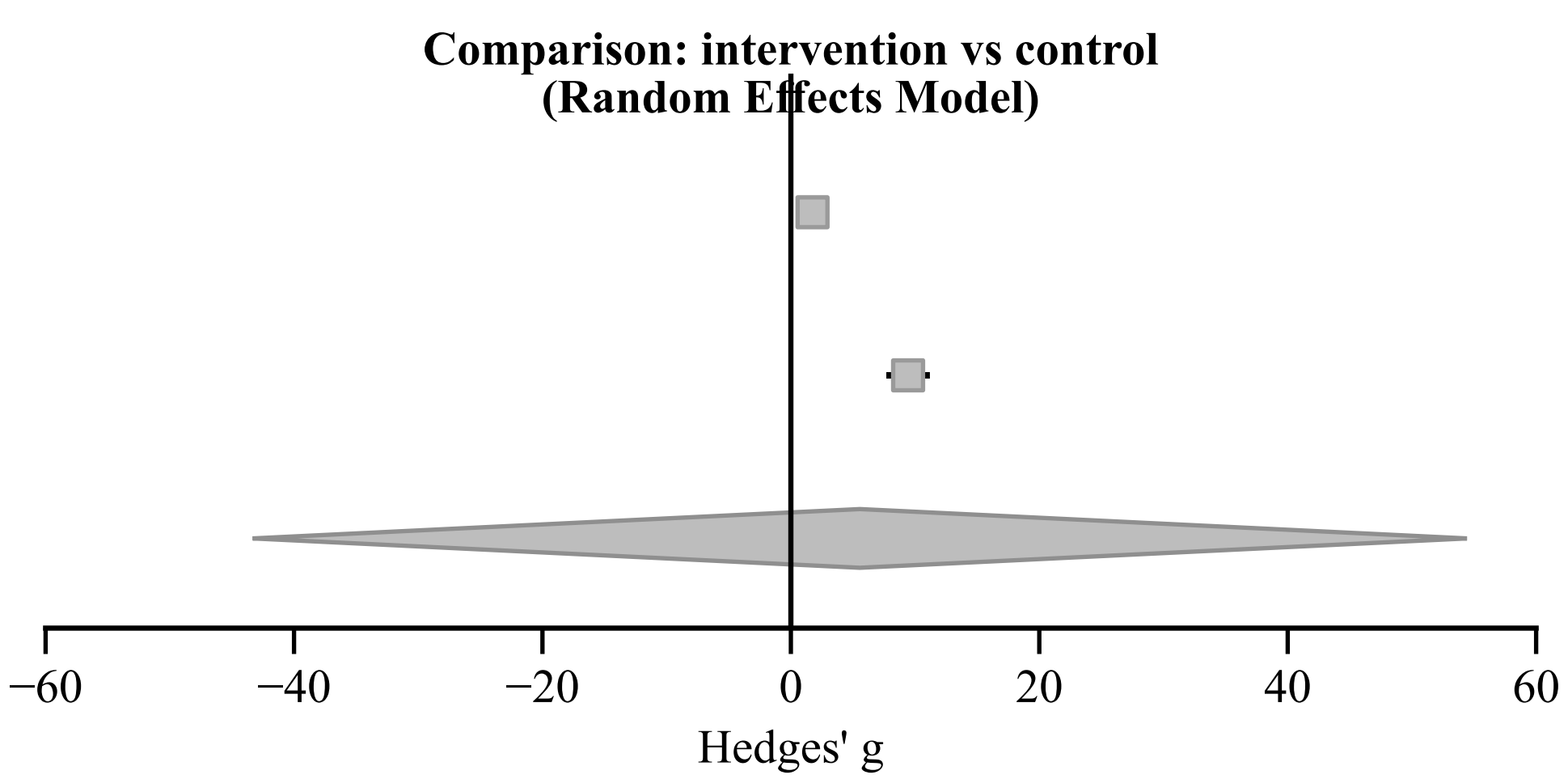


| Study | SMD | 95%-CI |
|---|---|---|
| Li 2022 | 1.74 | [1.41; 2.08] |
| Mobasheri 2022 | 9.44 | [7.94; 10.93] |
| **Pooled** | **5.55** | **[-43.32; 54.43]** |
