# Supplementary material for: Behavioral motivation support versus personalized dietary management for metabolic and behavioral outcomes in MASLD: a network meta-analysis
Source: Open Med (Wars). 2026 Sep 29;21(1):20261542. doi: 10.1515/med-2026-1542 (PMC13621874; doi:10.1515/med-2026-1542)
Supplement: Supplementary file 2 [file j_med-2026-1542_suppl_001.docx]

Supplementary File S1. PRISMA 2020 Checklist

Behavioral Motivation Support versus Personalized Dietary Management for Metabolic and Behavioral Outcomes in MASLD: A Network Meta-Analysis

Completed for the current revised manuscript. Page numbers refer to the revised manuscript PDF; supplementary-file labels refer to the files in the current delivery package.

| Section and Topic | Item # | Checklist Item | Location where item is reported |
| --- | --- | --- | --- |
| **TITLE** | | | |
| Title | 1 | Identify the report as a systematic review. | Title, p. 1 (identified as a network meta-analysis). |
| **ABSTRACT** | | | |
| Abstract | 2 | See the PRISMA 2020 for Abstracts checklist. | Abstract, p. 1. |
| **INTRODUCTION** | | | |
| Rationale | 3 | Describe the rationale for the review in the context of existing knowledge. | Introduction, pp. 1-2. |
| Objectives | 4 | Provide an explicit statement of the objective(s) or question(s) the review addresses. | Introduction, p. 2 (final paragraph). |
| **METHODS** | | | |
| Eligibility criteria | 5 | Specify the inclusion and exclusion criteria for the review and how studies were grouped for the syntheses. | Methods, Sections 2.1-2.2, pp. 2-3; Supplementary Table S6. |
| Information sources | 6 | Specify all databases, registers, websites, organisations, reference lists and other sources searched or consulted to identify studies. Specify the date when each source was last searched or consulted. | Methods, Section 2.1, pp. 2-3; Supplementary Table S1 (sources and final search dates). |
| Search strategy | 7 | Present the full search strategies for all databases, registers and websites, including any filters and limits used. | Supplementary Table S1 (complete strategies, dates, filters, limits, and source counts). |
| Selection process | 8 | Specify the methods used to decide whether a study met the inclusion criteria for the review, including how many reviewers screened each record and each report retrieved, whether they worked independently, and if applicable, details of automation tools used in the process. | Methods, Section 2.1, p. 3. |
| Data collection process | 9 | Specify the methods used to collect data from reports, including how many reviewers collected data from each report, whether they worked independently, any processes for obtaining or confirming data from study investigators, and if applicable, details of automation tools used in the process. | Methods, Section 2.1, p. 3 (pre-piloted form and extracted variables). |
| Data items | 10a | List and define all outcomes for which data were sought. Specify whether all results that were compatible with each outcome domain in each study were sought, and if not, the methods used to decide which results to collect. | Introduction, p. 2; Methods, Section 2.3, pp. 3-4. |
| Data items | 10b | List and define all other variables for which data were sought. Describe any assumptions made about any missing or unclear information. | Methods, Sections 2.1-2.3, pp. 2-4; Supplementary Tables S2 and S6. |
| Study risk of bias assessment | 11 | Specify the methods used to assess risk of bias in the included studies, including details of the tool(s) used, how many reviewers assessed each study and whether they worked independently, and if applicable, details of automation tools used in the process. | Methods, Section 2.3, pp. 3-4; Supplementary Table S7. |
| Effect measures | 12 | Specify for each outcome the effect measure(s) used in the synthesis or presentation of results. | Methods, Section 2.3, p. 3 (MD, Hedges' g, and natural-scale OR, each with 95% CI). |
| Synthesis methods | 13a | Describe the processes used to decide which studies were eligible for each synthesis. | Methods, Sections 2.1-2.3, pp. 2-4; Supplementary Table S6. |
| Synthesis methods | 13b | Describe any methods required to prepare the data for presentation or synthesis, such as handling of missing summary statistics, or data conversions. | Methods, Section 2.3, pp. 3-4; Supplementary Table S5. |
| Synthesis methods | 13c | Describe any methods used to tabulate or visually display results of individual studies and syntheses. | Figures 1-5; Tables 1-3; Supplementary Tables S2-S10 and Supplementary Figure S27. |
| Synthesis methods | 13d | Describe any methods used to synthesize results and provide a rationale for the choice(s). If meta-analysis was performed, describe the model(s), method(s) to identify the presence and extent of statistical heterogeneity, and software package(s) used. | Methods, Section 2.3, pp. 3-4. |
| Synthesis methods | 13e | Describe any methods used to explore possible causes of heterogeneity among study results. | Methods, Section 2.3, p. 4; Results, Sections 3.5-3.6, pp. 10-12; Supplementary Tables S2 and S9. |
| Synthesis methods | 13f | Describe any sensitivity analyses conducted to assess robustness of the synthesized results. | Methods, Section 2.3, p. 4; Results, Sections 3.5-3.6, pp. 10-12; Supplementary Tables S5 and S9; Supplementary Figure S27. |
| Reporting bias assessment | 14 | Describe any methods used to assess risk of bias due to missing results in a synthesis. | Methods, Section 2.3, p. 4; Results, Sections 3.5-3.6, pp. 10-12; Supplementary Table S10. |
| Certainty assessment | 15 | Describe any methods used to assess certainty (or confidence) in the body of evidence for an outcome. | Methods, Section 2.3, p. 4; Results, Section 3.6, pp. 10-12; Table 3; Supplementary Table S10. |
| **RESULTS** | | | |
| Study selection | 16a | Describe the results of the search and selection process, from the number of records identified in the search to the number of studies included in the review, ideally using a flow diagram. | Results, Section 3.1, pp. 4-5; Figure 1. |
| Study selection | 16b | Cite studies that might appear to meet the inclusion criteria, but which were excluded, and explain why they were excluded. | Results, Section 3.1, pp. 4-5; Supplementary Table S8 (all 92 exclusions and reasons). |
| Study characteristics | 17 | Cite each included study and present its characteristics. | Results, Section 3.2, pp. 5-7; Table 1; Supplementary Table S6. |
| Risk of bias in studies | 18 | Present assessments of risk of bias for each included study. | Results, Section 3.3, p. 7; Figure 2; Supplementary Table S7. |
| Results of individual studies | 19 | For all outcomes, present, for each study: (a) summary statistics for each group and (b) an effect estimate and its precision, ideally using structured tables or plots. | Results, Section 3.5, pp. 8-10; Table 2; Supplementary Tables S3-S6 and S9. |
| Results of syntheses | 20a | For each synthesis, briefly summarise the characteristics and risk of bias among contributing studies. | Results, Sections 3.2-3.4, pp. 5-7; Supplementary Tables S2, S6, and S7. |
| Results of syntheses | 20b | Present results of all statistical syntheses conducted. If meta-analysis was done, present for each the summary estimate and its precision and measures of statistical heterogeneity. If comparing groups, describe the direction of the effect. | Results, Section 3.5, pp. 8-10; Table 2; Supplementary Tables S3-S5. |
| Results of syntheses | 20c | Present results of all investigations of possible causes of heterogeneity among study results. | Results, Sections 3.5-3.6, pp. 8-12; Supplementary Tables S2 and S9. |
| Results of syntheses | 20d | Present results of all sensitivity analyses conducted to assess the robustness of the synthesized results. | Results, Sections 3.5-3.6, pp. 8-12; Supplementary Tables S5 and S9; Supplementary Figure S27. |
| Reporting biases | 21 | Present assessments of risk of bias due to missing results for each synthesis assessed. | Results, Sections 3.5-3.6, pp. 10-12; Supplementary Table S10 (reporting-bias domain); small-study-effect limitations are reported on p. 10. |
| Certainty of evidence | 22 | Present assessments of certainty (or confidence) in the body of evidence for each outcome assessed. | Results, Section 3.6, pp. 10-12; Figure 5D; Table 3; Supplementary Table S10. |
| **DISCUSSION** | | | |
| Discussion | 23a | Provide a general interpretation of the results in the context of other evidence. | Discussion, pp. 12-13. |
| Discussion | 23b | Discuss any limitations of the evidence included in the review. | Discussion, pp. 12-13. |
| Discussion | 23c | Discuss any limitations of the review processes used. | Discussion, pp. 12-13 (registration timing, reconstructed assessments, sparse networks, and underpowered small-study-effect assessment). |
| Discussion | 23d | Discuss implications of the results for practice, policy, and future research. | Discussion, pp. 12-13; Conclusions, p. 13. |
| **OTHER INFORMATION** | | | |
| Registration and protocol | 24a | Provide registration information for the review, including register name and registration number, or state that the review was not registered. | Abstract, p. 1; Methods opening paragraph, p. 2 (PROSPERO CRD420261371973; first submitted 17 April and registered 18 April 2026). |
| Registration and protocol | 24b | Indicate where the review protocol can be accessed, or state that a protocol was not prepared. | Methods opening paragraph, p. 2 (PROSPERO record CRD420261371973, version 1.0). |
| Registration and protocol | 24c | Describe and explain any amendments to information provided at registration or in the protocol. | No protocol amendment is reported as applicable; registration timing and the non-prospective status are disclosed in Methods, p. 2, and Discussion, pp. 12-13. |
| Support | 25 | Describe sources of financial or non-financial support for the review, and the role of the funders or sponsors in the review. | Funding statement, p. 13 (Science and Technology Key Project of Henan Province, grant 262102311136). |
| Competing interests | 26 | Declare any competing interests of review authors. | Conflicts of Interest statement, p. 13. |
| Availability of data, code and other materials | 27 | Report which of the following are publicly available and where they can be found: template data collection forms; data extracted from included studies; data used for all analyses; analytic code; any other materials used in the review. | Data Availability Statement, p. 13; data and supporting materials are included in the article and Supplementary Information. |

**From:** Page MJ, McKenzie JE, Bossuyt PM, et al. The PRISMA 2020 statement: an updated guideline for reporting systematic reviews. BMJ 2021;372:n71. doi: 10.1136/bmj.n71
