# Supplementary material for: Behavioral motivation support versus personalized dietary management for metabolic and behavioral outcomes in MASLD: a network meta-analysis
Source: Open Med (Wars). 2026 Sep 29;21(1):20261542. doi: 10.1515/med-2026-1542 (PMC13621874; doi:10.1515/med-2026-1542)
Supplement: Supplementary file 3 [file j_med-2026-1542_suppl_005.docx]

# Search Strategy

Topic: Behavioral motivation support and personalized dietary management for metabolic and behavioral outcomes in adults with MASLD.

Search period: Database inception to 31 March 2026 (final search date: 31 March 2026).

Sources: Eight bibliographic databases and two trial registers; no language restriction.

## 1. PubMed

| Module | Search # | Search Strategy | Period |
| --- | --- | --- | --- |
| Population | #1 | "Non-alcoholic Fatty Liver Disease"[Mesh] OR MASLD[tiab] OR "metabolic dysfunction-associated steatotic liver disease"[tiab] OR "metabolic dysfunction associated steatotic liver disease"[tiab] OR MAFLD[tiab] OR "metabolic dysfunction-associated fatty liver disease"[tiab] OR "metabolic dysfunction associated fatty liver disease"[tiab] OR NAFLD[tiab] OR "nonalcoholic fatty liver disease"[tiab] OR "non-alcoholic fatty liver disease"[tiab] OR MASH[tiab] OR NASH[tiab] OR "nonalcoholic steatohepatitis"[tiab] OR "non-alcoholic steatohepatitis"[tiab] OR "metabolic dysfunction-associated steatohepatitis"[tiab] OR "steatotic liver disease"[tiab] OR (("fatty liver"[tiab] OR "hepatic steatosis"[tiab] OR "liver steatosis"[tiab]) AND (nonalcoholic[tiab] OR "non-alcoholic"[tiab] OR "metabolic dysfunction"[tiab])) | Inception-Mar 31 2026 |
| Personalized Dietary Management | #2 | "Nutrition Therapy"[Mesh] OR "Diet Therapy"[Mesh] OR "Caloric Restriction"[Mesh] OR "Diet, Mediterranean"[Mesh] OR "dietary intervention*"[tiab] OR "nutrition intervention*"[tiab] OR "dietary management"[tiab] OR "diet management"[tiab] OR "dietary counseling"[tiab] OR "dietary counselling"[tiab] OR "nutrition counseling"[tiab] OR "nutrition counselling"[tiab] OR "medical nutrition therapy"[tiab] OR "personalized nutrition"[tiab] OR "personalised nutrition"[tiab] OR "precision nutrition"[tiab] OR "individualized nutrition"[tiab] OR "individualised nutrition"[tiab] OR "tailored nutrition"[tiab] OR "personalized diet*"[tiab] OR "personalised diet*"[tiab] OR "individualized diet*"[tiab] OR "individualised diet*"[tiab] OR "tailored diet*"[tiab] OR "customized diet*"[tiab] OR "customised diet*"[tiab] OR "meal plan*"[tiab] OR "hypocaloric diet"[tiab] OR "energy restriction"[tiab] OR "calorie restriction"[tiab] | Inception-Mar 31 2026 |
| Behavioral Motivation / Adherence Support | #3 | "Motivational Interviewing"[Mesh] OR "Behavior Therapy"[Mesh] OR "Patient Compliance"[Mesh] OR "Health Behavior"[Mesh] OR "Counseling"[Mesh] OR "motivational interviewing"[tiab] OR "motivational enhancement"[tiab] OR "behavior change"[tiab] OR "behaviour change"[tiab] OR "behavior modification"[tiab] OR "behaviour modification"[tiab] OR "behavioral counseling"[tiab] OR "behavioural counselling"[tiab] OR "behavioral support"[tiab] OR "behavioural support"[tiab] OR "self-monitoring"[tiab] OR "self monitoring"[tiab] OR "goal setting"[tiab] OR coaching[tiab] OR "health coaching"[tiab] OR adherence[tiab] OR complianc*[tiab] OR "patient adherence"[tiab] OR "dietary adherence"[tiab] OR "treatment adherence"[tiab] OR "behavioral intervention*"[tiab] OR "behavioural intervention*"[tiab] | Inception-Mar 31 2026 |
| RCT Filter (Cochrane HSSS adapted) | #4 | "Randomized Controlled Trial"[pt] OR "Controlled Clinical Trial"[pt] OR randomized[tiab] OR randomised[tiab] OR randomly[tiab] OR placebo[tiab] OR trial[tiab] OR groups[tiab] | - |
| Animal Exclusion | #5 | animals[mh] NOT humans[mh] | - |
| Final Combination | #6 | #1 AND (#2 OR #3) AND #4 NOT #5 | Inception-Mar 31 2026 |

## 2. Embase (via Embase.com)

| Module | Search # | Search Strategy | Period |
| --- | --- | --- | --- |
| Population | #1 | 'nonalcoholic fatty liver disease'/exp OR masld:ti,ab OR 'metabolic dysfunction-associated steatotic liver disease':ti,ab OR 'metabolic dysfunction associated steatotic liver disease':ti,ab OR mafld:ti,ab OR 'metabolic dysfunction-associated fatty liver disease':ti,ab OR 'metabolic dysfunction associated fatty liver disease':ti,ab OR nafld:ti,ab OR 'nonalcoholic fatty liver disease':ti,ab OR 'non-alcoholic fatty liver disease':ti,ab OR mash:ti,ab OR nash:ti,ab OR 'nonalcoholic steatohepatitis':ti,ab OR 'non-alcoholic steatohepatitis':ti,ab OR 'metabolic dysfunction-associated steatohepatitis':ti,ab OR 'steatotic liver disease':ti,ab OR (('fatty liver':ti,ab OR 'hepatic steatosis':ti,ab OR 'liver steatosis':ti,ab) AND (nonalcoholic:ti,ab OR 'non-alcoholic':ti,ab OR 'metabolic dysfunction':ti,ab)) | Inception-Mar 31 2026 |
| Personalized Dietary Management | #2 | 'nutrition therapy'/exp OR 'diet therapy'/exp OR 'calorie restriction'/exp OR 'mediterranean diet'/exp OR 'dietary intervention*':ti,ab OR 'nutrition intervention*':ti,ab OR 'dietary management':ti,ab OR 'diet management':ti,ab OR 'dietary counseling':ti,ab OR 'dietary counselling':ti,ab OR 'nutrition counseling':ti,ab OR 'nutrition counselling':ti,ab OR 'medical nutrition therapy':ti,ab OR 'personalized nutrition':ti,ab OR 'personalised nutrition':ti,ab OR 'precision nutrition':ti,ab OR 'individualized nutrition':ti,ab OR 'individualised nutrition':ti,ab OR 'tailored nutrition':ti,ab OR 'personalized diet*':ti,ab OR 'personalised diet*':ti,ab OR 'individualized diet*':ti,ab OR 'individualised diet*':ti,ab OR 'tailored diet*':ti,ab OR 'customized diet*':ti,ab OR 'customised diet*':ti,ab OR 'meal plan*':ti,ab OR 'hypocaloric diet':ti,ab OR 'energy restriction':ti,ab OR 'calorie restriction':ti,ab | Inception-Mar 31 2026 |
| Behavioral Motivation / Adherence Support | #3 | 'motivational interviewing'/exp OR 'behavior therapy'/exp OR 'patient compliance'/exp OR 'health behavior'/exp OR 'counseling'/exp OR 'motivational interviewing':ti,ab OR 'motivational enhancement':ti,ab OR 'behavior change':ti,ab OR 'behaviour change':ti,ab OR 'behavior modification':ti,ab OR 'behaviour modification':ti,ab OR 'behavioral counseling':ti,ab OR 'behavioural counselling':ti,ab OR 'behavioral support':ti,ab OR 'behavioural support':ti,ab OR 'self-monitoring':ti,ab OR 'self monitoring':ti,ab OR 'goal setting':ti,ab OR coaching:ti,ab OR 'health coaching':ti,ab OR adherence:ti,ab OR complianc*:ti,ab OR 'patient adherence':ti,ab OR 'dietary adherence':ti,ab OR 'treatment adherence':ti,ab OR 'behavioral intervention*':ti,ab OR 'behavioural intervention*':ti,ab | Inception-Mar 31 2026 |
| RCT Filter (Cochrane adapted) | #4 | 'randomized controlled trial'/exp OR 'controlled clinical trial'/exp OR 'clinical trial'/exp OR random*:ti,ab OR placebo:ti,ab OR trial:ti,ab OR groups:ti,ab OR 'double blind':ti,ab OR 'single blind':ti,ab | - |
| Exclusions | #5 | 'editorial'/it OR 'letter'/it OR 'note'/it | - |
| Final Combination | #6 | #1 AND (#2 OR #3) AND #4 NOT #5 | Inception-Mar 31 2026 |

## 3. Cochrane Library (CENTRAL)

| Module | Search # | Search Strategy | Period |
| --- | --- | --- | --- |
| Population | #1 | MeSH descriptor: [Non-alcoholic Fatty Liver Disease] explode all trees OR (MASLD OR "metabolic dysfunction-associated steatotic liver disease" OR "metabolic dysfunction associated steatotic liver disease" OR MAFLD OR "metabolic dysfunction-associated fatty liver disease" OR "metabolic dysfunction associated fatty liver disease" OR NAFLD OR "nonalcoholic fatty liver disease" OR "non-alcoholic fatty liver disease" OR MASH OR NASH OR "nonalcoholic steatohepatitis" OR "non-alcoholic steatohepatitis" OR "metabolic dysfunction-associated steatohepatitis" OR "steatotic liver disease" OR (("fatty liver" OR "hepatic steatosis" OR "liver steatosis") AND (nonalcoholic OR "non-alcoholic" OR "metabolic dysfunction"))):ti,ab,kw | Inception-Mar 31 2026 |
| Personalized Dietary Management | #2 | MeSH descriptor: [Nutrition Therapy] explode all trees OR MeSH descriptor: [Diet Therapy] explode all trees OR MeSH descriptor: [Caloric Restriction] explode all trees OR MeSH descriptor: [Diet, Mediterranean] explode all trees OR ("dietary intervention*" OR "nutrition intervention*" OR "dietary management" OR "diet management" OR "dietary counseling" OR "dietary counselling" OR "nutrition counseling" OR "nutrition counselling" OR "medical nutrition therapy" OR "personalized nutrition" OR "personalised nutrition" OR "precision nutrition" OR "individualized nutrition" OR "individualised nutrition" OR "tailored nutrition" OR "personalized diet*" OR "personalised diet*" OR "individualized diet*" OR "individualised diet*" OR "tailored diet*" OR "customized diet*" OR "customised diet*" OR "meal plan*" OR "hypocaloric diet" OR "energy restriction" OR "calorie restriction"):ti,ab,kw | Inception-Mar 31 2026 |
| Behavioral Motivation / Adherence Support | #3 | MeSH descriptor: [Motivational Interviewing] explode all trees OR MeSH descriptor: [Behavior Therapy] explode all trees OR MeSH descriptor: [Patient Compliance] explode all trees OR MeSH descriptor: [Health Behavior] explode all trees OR MeSH descriptor: [Counseling] explode all trees OR ("motivational interviewing" OR "motivational enhancement" OR "behavior change" OR "behaviour change" OR "behavior modification" OR "behaviour modification" OR "behavioral counseling" OR "behavioural counselling" OR "behavioral support" OR "behavioural support" OR "self-monitoring" OR "self monitoring" OR "goal setting" OR coaching OR "health coaching" OR adherence OR complianc* OR "patient adherence" OR "dietary adherence" OR "treatment adherence" OR "behavioral intervention*" OR "behavioural intervention*"):ti,ab,kw | Inception-Mar 31 2026 |
| Final Combination | #4 | #1 AND (#2 OR #3) - Limited to Trials | Inception-Mar 31 2026 |

Note: CENTRAL already focuses on controlled trials; therefore a separate RCT filter is generally not required.

## 4. Web of Science Core Collection

| Module | Search # | Search Strategy | Period |
| --- | --- | --- | --- |
| Population | #1 | TS=(MASLD OR "metabolic dysfunction-associated steatotic liver disease" OR "metabolic dysfunction associated steatotic liver disease" OR MAFLD OR "metabolic dysfunction-associated fatty liver disease" OR "metabolic dysfunction associated fatty liver disease" OR NAFLD OR "nonalcoholic fatty liver disease" OR "non-alcoholic fatty liver disease" OR MASH OR NASH OR "nonalcoholic steatohepatitis" OR "non-alcoholic steatohepatitis" OR "metabolic dysfunction-associated steatohepatitis" OR "steatotic liver disease" OR (("fatty liver" OR "hepatic steatosis" OR "liver steatosis") AND (nonalcoholic OR "non-alcoholic" OR "metabolic dysfunction"))) | Inception-Mar 31 2026 |
| Personalized Dietary Management | #2 | TS=("dietary intervention*" OR "nutrition intervention*" OR "dietary management" OR "diet management" OR "dietary counseling" OR "dietary counselling" OR "nutrition counseling" OR "nutrition counselling" OR "medical nutrition therapy" OR "personalized nutrition" OR "personalised nutrition" OR "precision nutrition" OR "individualized nutrition" OR "individualised nutrition" OR "tailored nutrition" OR "personalized diet*" OR "personalised diet*" OR "individualized diet*" OR "individualised diet*" OR "tailored diet*" OR "customized diet*" OR "customised diet*" OR "meal plan*" OR "hypocaloric diet" OR "energy restriction" OR "calorie restriction" OR "mediterranean diet") | Inception-Mar 31 2026 |
| Behavioral Motivation / Adherence Support | #3 | TS=("motivational interviewing" OR "motivational enhancement" OR "behavior change" OR "behaviour change" OR "behavior modification" OR "behaviour modification" OR "behavioral counseling" OR "behavioural counselling" OR "behavioral support" OR "behavioural support" OR "self-monitoring" OR "self monitoring" OR "goal setting" OR coaching OR "health coaching" OR adherence OR compliance OR "patient adherence" OR "dietary adherence" OR "treatment adherence" OR "behavioral intervention*" OR "behavioural intervention*") | Inception-Mar 31 2026 |
| RCT Filter | #4 | TS=(randomi* OR "controlled trial" OR "clinical trial" OR placebo OR "random* allocat*" OR "random* assign*" OR RCT OR "double blind" OR "single blind" OR trial OR groups) | - |
| Final Combination | #5 | #1 AND (#2 OR #3) AND #4 - Refined by: Document Types (Article OR Early Access OR Proceedings Paper) | Inception-Mar 31 2026 |

## 5. Scopus

| Module | Search # | Search Strategy | Period |
| --- | --- | --- | --- |
| Population | #1 | TITLE-ABS-KEY(MASLD OR "metabolic dysfunction-associated steatotic liver disease" OR "metabolic dysfunction associated steatotic liver disease" OR MAFLD OR "metabolic dysfunction-associated fatty liver disease" OR "metabolic dysfunction associated fatty liver disease" OR NAFLD OR "nonalcoholic fatty liver disease" OR "non-alcoholic fatty liver disease" OR MASH OR NASH OR "nonalcoholic steatohepatitis" OR "non-alcoholic steatohepatitis" OR "metabolic dysfunction-associated steatohepatitis" OR "steatotic liver disease" OR (("fatty liver" OR "hepatic steatosis" OR "liver steatosis") AND (nonalcoholic OR "non-alcoholic" OR "metabolic dysfunction"))) | Inception-Mar 31 2026 |
| Personalized Dietary Management | #2 | TITLE-ABS-KEY("dietary intervention*" OR "nutrition intervention*" OR "dietary management" OR "diet management" OR "dietary counseling" OR "dietary counselling" OR "nutrition counseling" OR "nutrition counselling" OR "medical nutrition therapy" OR "personalized nutrition" OR "personalised nutrition" OR "precision nutrition" OR "individualized nutrition" OR "individualised nutrition" OR "tailored nutrition" OR "personalized diet*" OR "personalised diet*" OR "individualized diet*" OR "individualised diet*" OR "tailored diet*" OR "customized diet*" OR "customised diet*" OR "meal plan*" OR "hypocaloric diet" OR "energy restriction" OR "calorie restriction" OR "mediterranean diet") | Inception-Mar 31 2026 |
| Behavioral Motivation / Adherence Support | #3 | TITLE-ABS-KEY("motivational interviewing" OR "motivational enhancement" OR "behavior change" OR "behaviour change" OR "behavior modification" OR "behaviour modification" OR "behavioral counseling" OR "behavioural counselling" OR "behavioral support" OR "behavioural support" OR "self-monitoring" OR "self monitoring" OR "goal setting" OR coaching OR "health coaching" OR adherence OR compliance OR "patient adherence" OR "dietary adherence" OR "treatment adherence" OR "behavioral intervention*" OR "behavioural intervention*") | Inception-Mar 31 2026 |
| RCT Filter | #4 | TITLE-ABS-KEY(randomi* OR "controlled trial" OR "clinical trial" OR placebo OR "random* allocat*" OR "random* assign*" OR RCT OR "double blind" OR "single blind" OR trial OR groups) | - |
| Document Type | #5 | DOCTYPE(ar) OR DOCTYPE(ip) OR DOCTYPE(cp) | - |
| Final Combination | #6 | #1 AND (#2 OR #3) AND #4 AND #5 | Inception-Mar 31 2026 |

## 6. ClinicalTrials.gov

**Access:** https://clinicaltrials.gov/

| Field | Search Terms |
| --- | --- |
| Condition or disease | MASLD OR MAFLD OR NAFLD OR MASH OR NASH OR "metabolic dysfunction associated steatotic liver disease" OR "nonalcoholic fatty liver disease" |
| Other terms | "personalized nutrition" OR "individualized diet" OR "dietary management" OR "medical nutrition therapy" OR "motivational interviewing" OR adherence OR compliance OR coaching OR lifestyle |
| Study type | Interventional Studies (Clinical Trials) |
| Status | All studies: completed, recruiting, active not recruiting, terminated, withdrawn, suspended |

## 7. WHO ICTRP

**Access:** https://trialsearch.who.int/

(MASLD OR MAFLD OR NAFLD OR MASH OR NASH OR "metabolic dysfunction associated steatotic liver disease" OR "nonalcoholic fatty liver disease") AND ("personalized nutrition" OR "dietary management" OR "motivational interviewing" OR adherence OR compliance OR coaching)

Manual screening is required because ICTRP aggregates multiple registries and keyword normalization is limited.

## 8. CNKI（专业检索）

**Access:** https://www.cnki.net/

**检索模式：**专业检索。

SU=(代谢相关脂肪性肝病 OR 代谢功能障碍相关脂肪性肝病 OR 代谢相关脂肪性肝炎 OR 非酒精性脂肪性肝病 OR 非酒精性脂肪肝 OR 非酒精性脂肪性肝炎 OR MASLD OR MAFLD OR NAFLD OR MASH OR NASH) AND SU=(个体化膳食 OR 个性化膳食 OR 个体化营养 OR 个性化营养 OR 个体化饮食 OR 个性化饮食 OR 营养治疗 OR 医学营养治疗 OR 饮食管理 OR 膳食管理 OR 膳食干预 OR 饮食干预 OR 动机性访谈 OR 动机访谈 OR 行为干预 OR 行为改变 OR 依从性 OR 依从性管理 OR 饮食依从性 OR 健康教练) AND SU=(随机 OR 对照 OR 随机对照试验 OR 临床试验 OR RCT)

## 9. Wanfang Data

**Access:** https://www.wanfangdata.com.cn/

**检索模式：**高级检索。为避免结果过宽，不使用全文字段限制随机词。

| Field | Chinese Terms |
| --- | --- |
| 题名或关键词 | 代谢相关脂肪性肝病 OR 代谢功能障碍相关脂肪性肝病 OR 非酒精性脂肪性肝病 OR 非酒精性脂肪肝 OR 非酒精性脂肪性肝炎 |
| AND 摘要 | 个体化膳食 OR 个性化营养 OR 个体化营养 OR 营养治疗 OR 医学营养治疗 OR 饮食管理 OR 膳食干预 OR 饮食干预 OR 动机性访谈 OR 行为干预 OR 依从性管理 |
| AND 摘要 | 随机 OR 对照 OR 随机对照试验 OR 临床试验 |

## 10. VIP Database

**Access:** http://www.cqvip.com/

**检索建议：**主题检索。

(代谢相关脂肪性肝病 OR 代谢功能障碍相关脂肪性肝病 OR 非酒精性脂肪性肝病 OR 非酒精性脂肪肝 OR 非酒精性脂肪性肝炎) AND (个体化膳食 OR 个性化营养 OR 营养治疗 OR 医学营养治疗 OR 饮食管理 OR 膳食干预 OR 动机性访谈 OR 行为干预 OR 依从性管理) AND (随机 OR 对照 OR 随机对照试验 OR 临床试验)

**Reference:** Lefebvre C, Glanville J, Briscoe S, Featherstone R, Littlewood A, Metzendorf MI, Noel-Storr A, Paynter R, Rader T, Thomas J, Wieland LS. Chapter 4: Searching for and selecting studies. In: Higgins JPT, Thomas J, Chandler J, Cumpston M, Li T, Page MJ, Welch VA, editors. Cochrane Handbook for Systematic Reviews of Interventions, Version 6.5. Cochrane, 2024. Available from Cochrane Handbook current website.

**Additional terminology source:** Rinella ME, Lazarus JV, Ratziu V, et al. A multisociety Delphi consensus statement on new fatty liver disease nomenclature. Hepatology. 2023.

Abbreviations: MeSH = Medical Subject Headings; tiab = title/abstract; ti,ab,kw = title, abstract, keywords; pt = publication type; exp = explode; TS = topic search; RCT = randomized controlled trial; MASLD = metabolic dysfunction-associated steatotic liver disease; MAFLD = metabolic dysfunction-associated fatty liver disease; NAFLD = nonalcoholic fatty liver disease; MASH = metabolic dysfunction-associated steatohepatitis; NASH = nonalcoholic steatohepatitis.

Note: As of MeSH 2026, the controlled vocabulary heading in PubMed remains "Non-alcoholic Fatty Liver Disease"; therefore both current nomenclature (MASLD/MASH) and historical terminology (NAFLD/NASH/MAFLD) were retained to maximize sensitivity.

Note: Conference records were not excluded a priori from Embase and can also be captured in Web of Science/Scopus where needed; final eligibility should be determined during screening according to the review protocol.

**Search execution log and source-count reconciliation**

| **Source** | **Type** | **Final search date** | **Records** | **Documentation note** |
| --- | --- | --- | --- | --- |
| PubMed | Database | 31 March 2026 | 623 | Export retained in source-level count |
| Embase | Database | 31 March 2026 | 4,837 | Export retained in source-level count |
| Scopus | Database | 31 March 2026 | 784 | Export retained in source-level count |
| Web of Science | Database | 31 March 2026 | 965 | Export retained in source-level count |
| Wanfang Data | Database | 31 March 2026 | 1,630 | Export retained in source-level count |
| Cochrane CENTRAL | Database | 31 March 2026 | 1,230 | Export retained in source-level count |
| CNKI | Database | 31 March 2026 | 0 | Search completed; zero records; no citation export created |
| VIP | Database | 31 March 2026 | 0 | Search completed; zero records; no citation export created |
| ClinicalTrials.gov | Register | 31 March 2026 | 25 | Registry records |
| WHO ICTRP | Register | 31 March 2026 | 34 | Registry records |
| Total | All sources | 31 March 2026 | 10,128 | 10,069 database + 59 register records |

Zero-result documentation: CNKI and VIP were searched using the strategies shown above and returned zero records. Consequently, neither platform generated a citation-export file. A search-history screen or screenshot showing the search date, full query, and zero-result count should be retained as the primary evidence when available.

Record-level reconciliation: source-level counts total 10,128, whereas the retained import used for deduplication contains 8,905 record-level entries. The 1,223-record difference cannot be reconstructed from the retained files and is disclosed in the PRISMA diagram. After removal of 1,768 duplicates, 7,137 records were screened. The original analysis log records 7,031 title/abstract exclusions, 106 reports and registry records assessed for eligibility, 92 excluded, and 14 included. The final reconciled eligibility-stage ledger in Supplementary Table S8 itemizes all 92 exclusions with reasons and supersedes the earlier incomplete 51-record ledger.
