# Supplementary material for: Behavioral motivation support versus personalized dietary management for metabolic and behavioral outcomes in MASLD: a network meta-analysis
Source: Open Med (Wars). 2026 Sep 29;21(1):20261542. doi: 10.1515/med-2026-1542 (PMC13621874; doi:10.1515/med-2026-1542)
Supplement: Supplementary file 4 [file j_med-2026-1542_suppl_013.docx]

Supplementary Materials: Supplementary Table S1, complete search strategies, dates, and source counts; Supplementary Tables S2–S10, transitivity, secondary and sparse analyses, node mapping, result-level RoB 2 judgments, all 92 excluded reports and registry records with reasons, nutrition-behavior r = 0.3/0.5/0.7 sensitivity analyses in S5, Joshi-exclusion sensitivity analyses only in S9, and complete CINeMA assessments for 88 network comparisons plus GRADE in S10; Supplementary Figure S27, nutrition-behavior sensitivity analyses; Supplementary File S1, PRISMA 2020 checklist.
